# Association between maximum norepinephrine dosage and mortality risk in neonates with septic shock

**DOI:** 10.1038/s41598-024-65744-4

**Published:** 2024-06-27

**Authors:** Junjuan Zhong, Jing Zhang, Yingyi Lin, Dongju Ma, Jing Mo, Xiuzhen Ye

**Affiliations:** Department of Neonatology, Guangdong Women and Children Hospital, Guangzhou, China

**Keywords:** Septic shock, Norepinephrine, Mortality, Neonates, Paediatric research, Risk factors

## Abstract

The high-dose usage of norepinephrine is thought to cause high mortality in patients with septic shock. This study aims to explores the correlation between the maximum norepinephrine (NE) dosage (MND) and mortality in neonates with septic shock. This retrospective cohort study included neonates with evidence of septic shock and those who received NE infusion. The study included 123 neonates, with 106 in the survival group and 17 in the death group. The death group exhibited significantly lower birth weight (*p* = 0.022), 1-min Apgar score (*p* = 0.005), serum albumin (*p* < 0.001), and base excess (BE) (*p* = 0.001) levels, but higher lactate (LAC) levels (*p* = 0.009) compared to the survival group. MND demonstrated an ROC area under the curve of 0.775 (95% CI 0.63–0.92, *p* < 0.001) for predicting mortality, with an optimal threshold of 0.3 µg/(kg·min), a sensitivity of 82.4%, and a specificity of 75.5%. Multivariate logistic regression indicated that an MND > 0.3 µg/(kg·min) (OR, 12.08, 95% CI 2.28–64.01) was associated with a significantly higher mortality risk. Spearman rank correlation showed a positive correlation between MND and LAC (*r* = 0.252, *p* = 0.005), vasoactive-inotropic score (VIS) (*r* = 0.836, *p* < 0.001), and a negative correlation with BE (*r* = − 0.311, *p* = 0.001). MND > 0.3 µg/(kg min) is a useful predictive marker of mortality in neonatal septic shock.

## Introduction

Sepsis accounts for about one-third of global neonatal mortality, and the World Health Organization has reported neonatal sepsis as a major future global health threat^1,2^. Statistics show 2,824 cases of sepsis per 100,000 live births. Septic shock is the most critical stage of sepsis progression and requires vasopressor treatment to maintain blood pressure despite adequate fluid resuscitation; the condition incurs a mortality rate of up to 40%^3–5^. Early identification of infants at risk of mortality holds significance in medical resourse allocation and enables early intervention to improve the prognosis.

Recommended as the first-line vasoactive agent in the treatment of pediatric septic shock by the latest international management guidelines, norepinephrine (NE) is extensively used in clinical practice^6^. In recent years, results from adult studies revealed that the use of high-dose NE was associated with poor outcomes in septic shock^7,8^. Martin et al. reported that an NE dosage of 1ug/kg/min was associated with an ICU death rate of 90% and suggested that a dosage of NE greater than 1ug/kg/min was an independent factor associated with mortality in adults with septic shock^9^. However, the correlation between the NE dosage and prognosis in neonates with septic shock remains unclear. This study aimed to evaluate the correlation between NE dosage and neonatal septic shock mortality.

## Materials and methods

### Subjects

The study retrospectively analyzed neonates diagnosed with septic shock and treated with NE in the Neonatal Intensive Care Unit (NICU) of Guangdong Women and Children Hospital from January 1, 2019, to December 31, 2021. The diagnostic criteria for sepsis followed the guidelines of the expert consensus on the diagnosis and management of neonatal sepsis (version 2019) revised by the Chinese Medical Association^10^. The inclusion criteria were neonates with septic shock^6,11^ and NE treatment. The exclusion criteria were congenital heart diseases (except patent foramen ovale and non-hemodynamically significant patent ductus arteriosus), disabling congenital malformations, or suspected congenital metabolic diseases. The study was approved by the Guangdong Women and Children Hospital Research Ethical Committee (No. 202301008). Informed consent was obtained from all subjects and/or parents or their legal guardians for study participation. All methods were performed in accordance with the relevant guidelines and regulations.

### Methods

The study retrospectively collected data on gender, gestational age, birth weight, perinatal conditions, and initial laboratory results post-septic shock diagnosis, including serum albumin (ALB), base excess (BE), lactate (LAC), platelet count (PLT), and white blood cell count (WBC). The worst values of LAC, BE, cardiac index during shock, maximum dosage of vasopressor, vasoactive-inotrope score (VIS), and septic shock score (SSS) were also recorded and analyzed. The VIS was calculated as follows: VIS = (dobutamine + dopamine in mcg/kg/min) + (milrinone in mcg/kg/min) × 10 + (epinephrine + norepinephrine in mcg/kg/min) × 100 + (vasopressin in IU/kg/min) × 10,000. The bedside SSS (bSSS) refers to a scale ranging from 0 to 5 points, with 1 point attributed for a LAC > 8 mmol/L, 1 point for VIS > 200, and 3 points for the presence of severe cardiomyopathy, as defined by a cardiac index < 2.2L/min/m^2^ or a left ventricle ejection fraction < 25%. The computed SSS (cSSS) was calculated as follows: cSSS = 1.1^LAC^ + 1.001^VIS^ + 18 (in the presence of severe cardiomyopathy)^12^.

### Statistical analysis

Data were analyzed using SPSS 22.0. Normally distributed data were expressed as mean (standard deviation) and compared using two-sample *t*-tests; non-normally distributed data were expressed as median (interquartile range) and compared using the Mann–Whitney *U* test; categorical data were expressed as frequencies and percentages and compared using the Chi-square test. The receiver operating characteristic (ROC) curve was used to assess the optimal cutoff for maximum norepinephrine dosage (MND) to evaluate the risk of mortality in neonatal septic shock. The optimal cutoff was determined by identifying the point having the largest sum of sensitivity and specificity. The MND was then converted into categorical variables using this cutoff. The multivariate logistic regression analysis identified independent risk factors for mortality. After adjusting for confounding factors, the OR value of the correlation between MND > 0.3 µg/kg/min and mortality was analyzed by multivariate logistic regression. Spearman rank correlation analysis was performed to assess the correlation between MND and indicators of poor prognosis. In this study, a* P*-value < 0.05 was considered statistically significant.

### Ethics approval and consent to participate

The study was approved by the Guangdong Women and Children Hospital Research Ethical Committee (No. 202301008). Informed consent was obtained from all subjects and/or parents or their legal guardians for study participation. All methods were performed in accordance with the relevant guidelines and regulations.

## Results

### Perinatal conditions and laboratory results

**Figure 1:**
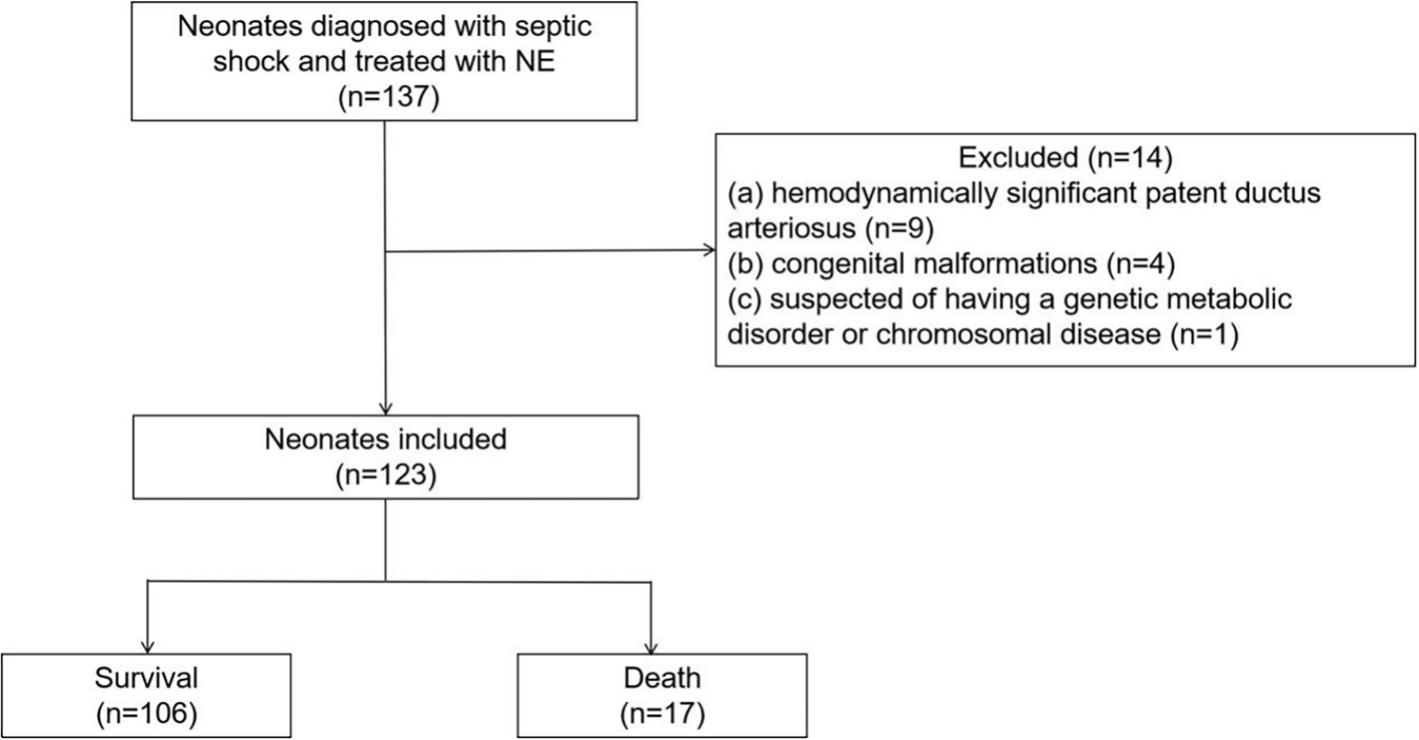
Study participant flow diagram.

Of the 137 neonates initially identified, 14 were excluded (nine with hemodynamically significant patent ductus arteriosus, four with congenital malformations, and one suspected of having a genetic metabolic disorder or chromosomal disease). Ultimately 123 neonates were included in the analysis, who were further divided into the survival (*n* = 106) and death (*n* = 17) groups. (Fig. 1).

The median time of onset of septic shock was 2 days after birth. A total of 12 neonates exhibited late-onset septic shock (9.8%). In addition, 21 out of the 123 infants had blood culture- positive sepsis. Compared to the survival group, the death group had a significantly lower birth weight (*p* = 0.022) and 1 min Apgar score (*p* = 0.005). (Table 1). In contrast, no significant differences were found in gestational age, gender, 5 min Apgar score, maternal pregnancy, and childbirth conditions (*p* > 0.05). The death group showed significantly lower ALB (*p* < 0.001) and BE (*p* = 0.001) levels but higher LAC (*p* = 0.009) levels after being diagnosed with septic shock. No significant differences in PLT, WBC < 5 × 10^9^/L, and positive blood culture rates were observed (*p* > 0.05). (Table 2).

**Table 1:** Descriptive perinatal characteristics of neonates with septic shock.

	Survival *N* = 106	Death *N* = 17	*P* value
Gestational age (weeks), median [IQR]	35.1 (30.3,38.0)	29.6 (28.0,37.0)	0.074
Birth weight (g), median [IQR]	2465 (1665,3200)	1250 (1030,1660)	0.022
Male infant	76 (71.7%)	8 (47.1%)	0.053
1 min Apgar score, median [IQR]	8 (7, 9)	6 (5,8)	0.005
5 min Apgar score, median [IQR]	9 (9,10)	9 (9,9)	0.142
Cesarean section	66 (62.3%)	66 (62.3%)	0.593
Maternal chorioamnionitis	6 (5.7%)	2 (11.8%)	0.676

IQR interquartile range

**Table 2:** Comparison of initial laboratory test results in neonates diagnosed with septic shock.

	Survival *N* = 106	Death *N* = 17	*P* value
Positive blood culture	15 (14.2%)	6 (35.3%)	0.075
WBC < 5 × 10^9^/L	19 (17.9%)	7 (41.2%)	0.063
PLT (× 10^9^/L), median [IQR]	230 (189,266)	138 (76,237)	0.083
ALB (g/L), mean/SD	26.3 (5.2)	19.1 (6.0)	< 0.001
LAC (mmol/L), median [IQR]	2.4 (1.8,3.8)	3.1 (1.9,6.9)	0.009
BE (mmol/L), mean/SD	− 4.9 (3.8)	− 8.5 (5.3)	0.001

WBC white blood cell count, PLT platelet count, IQR interquartile range, ALB serum albumin, SD standard deviation, LAC lactate, BE base excess.

### Predictive value of MND for mortality in neonates with septic shock

**Figure 2:**
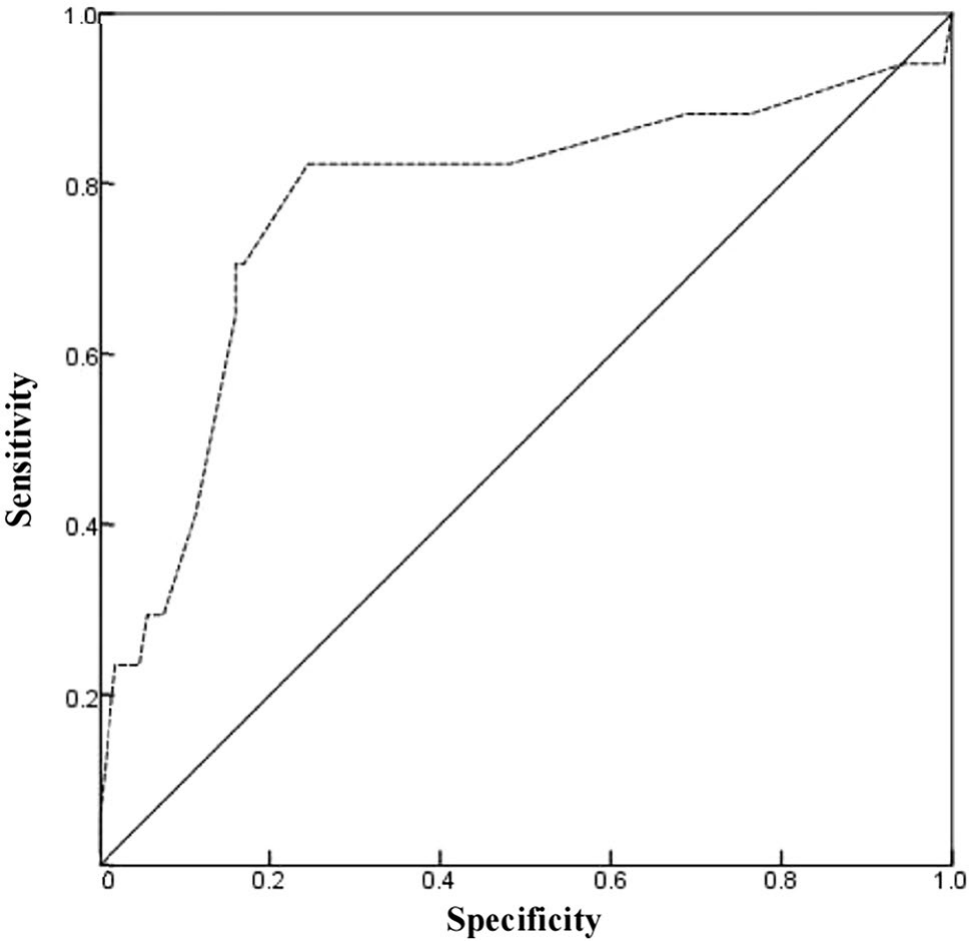
Receiver operating curve of maximum norepinephrine dosage. The area under the curve was 0.775 (95% CI: 0.63–0.92). A maximum norepinephrine dosage greater than 0.3 µg/kg/min predicted mortality with 82.4% sensitivity and 75.5% specificity.

The ROC curve for predicting neonatal septic shock mortality using MND showed an area under the curve of 0.775 (95% CI, 0.63–0.92, *p* < 0.001). The optimal threshold for MND was 0.3 µg/kg/min, with a sensitivity of 82.4%, specificity of 75.5%, positive predictive value of 82.4%, and negative predictive value of 75.5%. (Fig. 2).

### Multivariate logistic regression analysis for mortality in neonates with septic shock

Neonatal septic shock mortality was set as the dependent variable and birth weight, 1 min Apgar score, MND > 0.3 µg/kg/min, and initial levels of ALB, LAC, and BE were included as variables in the multivariate logistic regression analysis. The results indicated that MND > 0.3 µg/kg/min (OR, 12.08, 95% CI, 2.28–64.01) was an independent risk factor for mortality. (Table 3).

**Table 3:** Multivariate logistic regression analysis for mortality in neonates with septic shock.

	B	OR	95% CI	*P* value
Birth weight, g	− 0.001	1.00	0.99–1.00	0.147
1 min Apgar score	0.170	1.19	0.77–1.82	0.438
MND > 0.3ug/kg/min	2.492	12.08	2.28–64.01	0.003
ALB, g/L	− 0.207	0.81	0.66–1.00	0.053
LAC, mmol/L	0.139	1.15	0.92–1.43	0.216
BE, mmol/L	− 0.059	0.94	0.75–1.18	0.607

MND maximum norepinephrine dosage, ALB serum albumin, LAC lactate, BE base excess.

Correlation Analysis Between MND and Indicators of Poor Prognostic.

Spearman rank correlation analysis indicated that the worst values of LAC (*r* = 0.252, *p* = 0.005) and VIS (*r* = 0.836, *p* < 0.001) during septic shock were positively correlated with MND, whereas BE (*r* = -0.311, *p* = 0.001) was negatively correlated. No correlation was found with SSS (*p* > 0.05). (Table 4).

**Table 4:** Correlation between maximum norepinephrine dosage and poor prognostic indicators in neonates with septic shock.

Variables	Maximum norepinephrine dosage (ug/kg/min)	
	*r*	*p*
BE, mmol/L	− 0.311	0.001
LAC, mmol/L	0.252	0.005
VIS	0.836	< 0.001
Computed SSS	0.118	0.268
Bedside SSS	0.201	0.059

BE base excess, LAC lactate, VIS vasoactive-inotropic score, SSS septic shock score.

## Discussion

Despite advances in the understanding of the pathogenesis and therapeutic principles of neonatal septic shock, mortality rates remain high. Identifying of factors that contribute to an increased risk of mortality could aid in improving survival rates in neonates with septic shock. High doses of NE have been associated with mortality in adults with septic shock^7,13^, but the evidence supporting this association in children is limited. To the best of our knowledge, the present study is the first to demonstrate a significant correlation between MND > 0.3 µg/kg/min and neonatal septic shock mortality, showing high sensitivity, specificity, and predictive values for mortality prediction.

Current international guidelines for pediatric sepsis and septic shock recommend NE as the first-choice vasopressor for children but explicitly exclude preterm infants^6^. These guidelines acknowledge that neonates, compared to older children, may require different vasopressor support strategies in septic shock^14^. Until now, dopamine has been recommended as the first-line vasopressor for neonatal septic shock^11^. However, growing evidence suggests that dopamine, especially in high doses, may also raise the mortality and adverse event rates in shock patients^15^. Recently, NE has been increasingly used in neonatal septic shock. Studies have revealed that NE significantly improves cardiac output, blood pressure, organ perfusion, and urine output in neonates with septic shock while reducing the dosage of dopamine and shortening the duration of vasopressor use^16–18^. Nonetheless, high doses of NE may induce oxidative stress and myocardial cell insult^19^.

Recent studies demonstrate a significant independent correlation between high-dosage NE and mortality risk in septic shock adult patients^7,13^. Research indicates that NE dosages ≥ 0.6 µg/kg/min within 24 h are significantly related to 7-day mortality in septic shock patients, which is likely due to sympathetic overstimulation causing myocardial damage^7,20^. A previous study reported that every 10 µg/min increase in NE dosage let to a 20.7% increase in mortality^13^. The findings of our study suggest that MND > 0.3µg/kg/min is a significant independent predictor of mortality in neonates with septic shock. This threshold demonstrates high sensitivity, specificity, and positive and negative predictive values all exceeding 75% in forecasting mortality in neonates with septic shock. Furthermore, MND is associated with poor prognostic indicators of septic shock, including BE, LAC, and VIS.

### Limitations

Nevertheless, the limitations of the present study should be acknowledged. This single-center retrospective analysis of NE dosage was adjusted based on clinical decisions rather than a prospective protocol. Moreover, the small sample size for the death group (*n* = 17) was the primary limitation of this study. Hence, even statistically significant results should be interpreted with caution and should be validated by studies with larger sample sizes. Finally, the current study only collected the early and the worst indicators of patients and did not analyze vasopressor therapies other than NE, which may potentially impact the assessment of mortality.

## Conclusion

MND > 0.3 µg/kg/min is significantly associated with mortality in neonates with septic shock and can be used as a key predictive indicator.

## Data Availability

The datasets generated and/or analyzed dyring the study are available from the corresponding author upon reasonable request.

## Abbreviations

ALB: Serum albumin
BE: Base excess
IQR: Interquartile range
LAC: Lactate
MND: Maximum norepinephrine dosage
NE: Norepinephrine
NICU: Neonatal intensive care unit
PLT: Platelet count
ROC: Receiver operating characteristic
SSS: Septic shock score
SD: Standard deviation
VIS: Vasoactive-inotropic score
